# Antiviral Functions of Human Immunodeficiency Virus Type 1 (HIV-1)-Specific IgG Antibodies: Effects of Antiretroviral Therapy and Implications for Therapeutic HIV-1 Vaccine Design

**DOI:** 10.3389/fimmu.2017.00780

**Published:** 2017-07-04

**Authors:** Martyn A. French, M. Christian Tjiam, Laila N. Abudulai, Sonia Fernandez

**Affiliations:** ^1^School of Biomedical Sciences, University of Western Australia, Perth, WA, Australia; ^2^Medical School, University of Western Australia, Perth, WA, Australia; ^3^Department of Clinical Immunology, Royal Perth Hospital and PathWest Laboratory Medicine, Perth, WA, Australia

**Keywords:** human immunodeficiency virus type 1, IgG antibody function, IgG subclasses, fragment crystallizable receptors, antiretroviral therapy

## Abstract

Contemporary antiretroviral therapy (ART) is effective and tolerable for long periods of time but cannot eradicate human immunodeficiency virus type 1 (HIV-1) infection by either elimination of viral reservoirs or enhancement of HIV-1-specific immune responses. Boosting “protective” HIV-1-specific immune responses by active or passive immunization will therefore be necessary to control or eradicate HIV-1 infection and is currently the topic of intense investigation. Recently reported studies conducted in HIV patients and non-human primate (NHP) models of HIV-1 infection suggest that HIV-1-specific IgG antibody responses may contribute to the control of HIV-1 infection. However, production of IgG antibodies with virus neutralizing activity by vaccination remains problematic and while vaccine-induced natural killer cell-activating IgG antibodies have been shown to prevent the acquisition of HIV-1 infection, they may not be sufficient to control or eradicate established HIV-1 infection. It is, therefore, important to consider other functional characteristics of IgG antibody responses. IgG antibodies to viruses also mediate opsonophagocytic antibody responses against virions and capsids that enhance the function of phagocytic cells playing critical roles in antiviral immune responses, particularly conventional dendritic cells and plasmacytoid dendritic cells. Emerging evidence suggests that these antibody functions might contribute to the control of HIV-1 infection. In addition, IgG antibodies contribute to the intracellular degradation of viruses *via* binding to the cytosolic fragment crystallizable (Fc) receptor tripartite motif containing-21 (TRIM21). The functional activity of an IgG antibody response is influenced by the IgG subclass content, which affects binding to antigens and to Fcγ receptors on phagocytic cells and to TRIM21. The IgG subclass content and avidity of IgG antibodies is determined by germinal center (GC) reactions in follicles of lymphoid tissue. As HIV-1 infects cells in GCs and induces GC dysfunction, which may persist during ART, strategies for boosting HIV-1-specific IgG antibody responses should include early commencement of ART and possibly the use of particular antiretroviral drugs to optimize drug levels in lymphoid follicles. Finally, enhancing particular functions of HIV-1-specific IgG antibody responses by using adjuvants or cytokines to modulate the IgG subclass content of the antibody response might be investigated in NHP models of HIV-1 infection and during trials of therapeutic vaccines in HIV patients.

## Introduction

Long-term control of human immunodeficiency virus type 1 (HIV-1) infection by vaccine-induced immune responses is a widely sought after, but currently unachievable, goal of HIV cure research. An incomplete understanding of “protective” HIV-1-specific immune responses is the main obstacle to achieve this goal. While it has been clearly established that CD8^+^ T-cell responses against peptides of HIV-1 capsid proteins encoded by *Gag* (Gag proteins) can control HIV-1 replication (1–3), especially in HIV controllers, who control HIV-1 replication without antiretroviral therapy (ART) (4), evasion of CD8^+^ T-cell responses occurs in most individuals. Furthermore, it has not been possible to replicate these “protective” HIV-1-specific CD8^+^ T-cell responses by vaccination (5, 6). Recently, it has been shown that infusions of broadly neutralizing human monoclonal antibodies (hMAbs) to HIV-1 envelope (Env) antigens are capable of suppressing HIV-1 replication (7–9), accelerating the elimination of HIV-1-infected CD4^+^ T cells (10) and enhancing production of antibodies that neutralize HIV-1 (11). In addition, studies in macaques with simian immunodeficiency virus (SIV) infection have shown that infusions of naturally occurring acute phase IgG antibodies to SIV Env antigens enhanced SIV-specific CD8^+^ T-cell responses by increasing virus uptake in antigen-presenting cells (APCs) (12). Based on these observations, there is currently guarded optimism that vaccine-induced HIV-1-specific antibody responses might contribute to long-term control, and possibly eradication, of chronic HIV-1 infection (13, 14).

While substantial attention has been paid to understanding the characteristics of HIV-1 Env-specific IgG antibodies required to neutralize HIV-1 replication (14, 15), there is a growing acceptance that the concept of antibody-mediated neutralization of viruses needs to be reassessed (16) and that HIV-1 Env-specific IgG antibodies also exert functional effects other than direct neutralization (17). In addition, we have provided evidence that natural control of HIV-1 infection might be associated with plasmacytoid dendritic cell (pDC)-reactive opsonophagocytic IgG antibody responses against antigens present on HIV-1 capsids (18, 19).

The processes required for the production, functional diversification, and maintenance of HIV-1-specific IgG antibody responses in HIV patients receiving ART are likely to be different, in many aspects, to those required for the production of antibody responses that prevent acquisition of HIV-1 infection. Here, we discuss the characteristics of HIV-1-specific antibody responses that are likely to be required for long-term control of HIV-1 infection and in doing so, look beyond the “neutralizing/non-neutralizing antibody” paradigm as well as consider the effects of HIV-induced immunopathology and ART on those antibody responses.

## Control of Virus Infections by Systemic Antibody Responses

**Figure 1:**
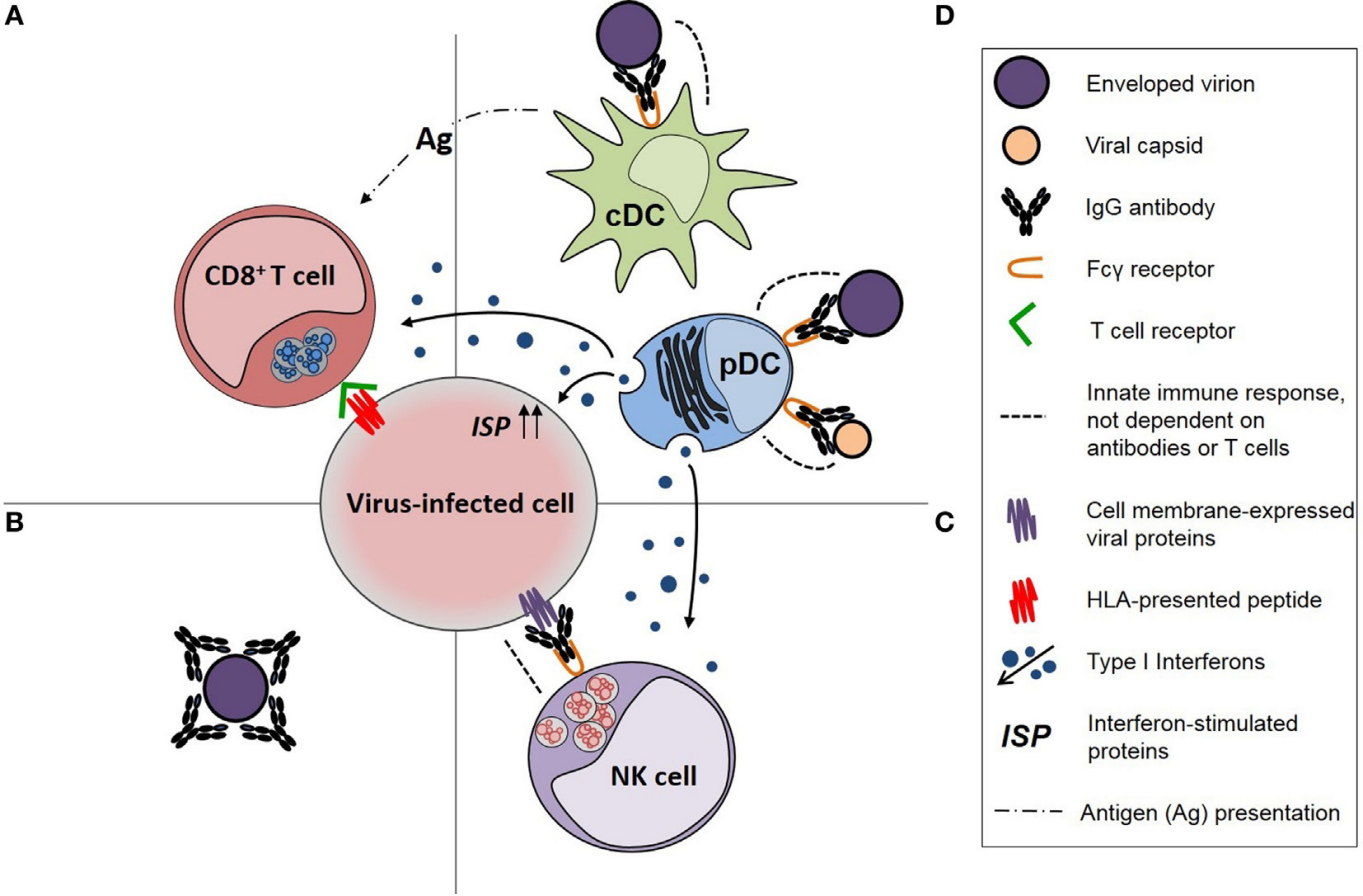
Diagrammatic representation of immune responses against viruses and the functional effects of virus-specific IgG antibodies in those responses. **(A)** CD8^+^ T-cell responses, which are dependent on the activity of antigen-presenting cells (APCs), **(B)** neutralizing antibodies, **(C)** natural killer (NK) cell responses, including activation by IgG antibodies, and **(D)** APC activity of conventional dendritic cells (cDCs) for CD8^+^ T cells and antiviral immune responses of plasmacytoid dendritic cell (pDCs), particularly production of type 1 interferons, both of which are enhanced by opsonophagocytic IgG antibody responses [modified from Tjiam et al. (18)].

Systemic antibody responses against viruses, including HIV-1, consist primarily of IgG antibodies, which are effective not only in the intravascular compartment but are also transported to interstitial, intercellular, and intracellular compartments of tissues and across the placenta facilitated by binding to the neonatal Fc receptor (FcRn) (20). Serum IgG antibodies also contribute to antibody responses on mucosal surfaces, possibly including IgG antibodies against HIV-1 V2 V3 Env glycans and the membrane proximal external region of HIV-1 gp41 (21). An IgG antibody response may exhibit pleiotropic functional effects (Table 1), but those of greatest relevance to “protective” antibody responses against viruses, including HIV-1, are (1) virus neutralization, (2) opsonization of viral particles and their phagocytosis by conventional dendritic cells (cDCs) and pDCs to enhance the antiviral effector functions of those cells, and (3) activation of natural killer (NK) cells to undertake antibody-dependent cellular cytotoxicity (ADCC; Figure 1). The importance of pDCs and NK cells in controlling virus infections is illustrated by the primary immunodeficiency disorders, such as DOCK8 deficiency and primary NK cell deficiency, in which deficiency of these cells is associated with an increased susceptibility to multiple virus infections (22, 23). Persistent virus infections are also a characteristic of primary immunodeficiency disorders in which cDC deficiency occurs (24). An IgG antibody response against a virus infection may also activate the complement pathway *via* C1q but, as exemplified by HIV-1 infection (25), the effects of complement activating antibodies may enhance virus infections rather than control them.

**Table 1:** Functional effects of an IgG antibody response.

Neutralization of microbial toxins and viruses
Opsonophagocytosis of antigens *via* FcγRI and/or FcγRIIa and FcγRIIb
Various phagocytic cells with differences in effector functions ○ Neutrophils and eosinophils ○ Monocytes/macrophages ○ Conventional dendritic cells ○ Plasmacytoid dendritic cells Various size of antigens ○ Fungi ○ Protozoans ○ Bacteria and mycobacteria ○ Virions of enveloped and non-enveloped viruses ○ Capsids of enveloped viruses?
Intracellular degradation of pathogens *via* tripartite motif containing-21, particularly viruses
Natural killer cell activation *via* FcγRIIc and FcγRIIIa
Eosinophil degranulation *via* FcγRIIa and FcγRIII
Complement system activation *via* C1q

### Shaping of IgG Antibody Responses in Germinal Centers (GCs) of Lymphoid Tissue

The functional effects of an IgG antibody response are shaped within GCs of follicles in secondary lymphoid tissues, including lymph nodes, spleen, and gut-associated lymphoid tissue. Here, immune complexes of antigen, IgG antibodies, and complement pathway molecules bind to receptors for the fragment crystallizable (Fc) domain of IgG [Fcγ receptors (FcγRs)] and complement [complement receptors (CRs)] on the surface of follicular dendritic cells (FDCs). Subsequent exposure of follicular B cells to antigens expressed by FDCs generates plasma cells and memory B cells with “help” from T follicular helper (T_FH_) cells. Within follicular B cells, somatic hypermutation (SHM) of genes encoding the variable region of immunoglobulin molecules, which constitute a part of the B cell receptor (BCR), give rise to follicular B cells expressing BCRs with various antigen-binding affinities. Deletion of B-cell clones expressing low affinity BCRs for antigens and differentiation of the remaining B cells into plasma cells results in the production of high-affinity antibodies, a process known as affinity maturation (26). The avidity (functional affinity) with which IgG antibodies bind to an antigen reflects the sum of antibody affinities for different epitopes on an antigen but is also affected by other factors, including valency of both the antibody and antigen epitopes. Persistent exposure of follicular B cells to antigens on FDCs is critical for maintaining memory B cells (27), in part through the maintenance of antigen-specific T_FH_ cells, the promotion of SHM, and the affinity maturation of GC B cells (28), and GC T_FH_ cell development (29, 30).

The GC reaction also exerts a dominant effect on the functional diversification of IgG antibody responses by providing a milieu in which follicular B cells expressing BCRs of one of the four IgG subclasses (IgG1–4) differentiate into memory B cells or plasma cells producing antibodies of that IgG subclass. Each IgG subclass exhibits differences in the structure and function of the hinge and Fc domain of the IgG molecule that confers variation in antibody function (Table 2). The heavy chain of IgG molecules, including the hinge region and Fc domains, is encoded by one of the four γ heavy chain genes, which are ordered 5′-γ3-γ1-γ2-γ4-3′, in the immunoglobulin heavy chain gene complex on chromosome 14 (31). The subclass of IgG expressed by a B cell can switch from IgG3 to one of the other three subclasses by a process of class switch recombination of γ heavy chain genes. Production of IgG3 and IgG1 occurs earlier than the production of IgG2 and IgG4 during the GC reaction, though it remains unclear if switching from one IgG subclass to another occurs sequentially or not (32, 33).

**Table 2:** Characteristics of IgG subclasses that affect antibody function.

	IgG3	IgG1	IgG2	IgG4
Antigen binding	High flexibility of hinge region, which is about four times longer than the equivalent region in other subclasses (34)		Opsonization of antigens with a high epitope density (35) Conformational variation resulting from rearrangement of disulfide bonding of the hinge region with the light and heavy chains (37, 38)	Monovalent bispecific antibodies resulting from exchange of half molecules between IgG molecules (36)

Effector function	Potent complement activation and binding to all Fcγ receptors (FcγRs) as monomers or multimers (39) Lower affinity binding to tripartite motif containing-21 than other IgG subclasses (39)	Complement activation and binding to all FcγRs as multimers only (39)	Low complement activation, sufficient for opsonophagocytosis of bacteria (40) Binding only to “low affinity” FcγRs, particularly FcγRIIa (39)	Absent complement activation. Binding to all FcγRs but with lower affinity than for IgG3 or IgG1 (39)

Other	Substantial genetic variation (13 immunoglobulin Gm allotypes) Shorter half-life (7 days) than other IgG subclasses (21 days) Least resistant to human proteolytic enzymes (cathepsin G) (42)	Moderate genetic variation (4 immunoglobulin Gm allotypes)	Low genetic variation (1 immunoglobulin Gm allotype) Covalent dimerization (41) Most resistant to human proteolytic enzymes (cathepsin G) (42)	No genetic variation (no immunoglobulin Gm allotypes)

Maturation of an IgG antibody response during a GC reaction is critically dependent on regulation of follicular B-cell differentiation by CD4^+^ T cells of the T_FH_ cell lineage (43–45). T_FH_ cells express high levels of the chemokine receptor CXCR5, which facilitates their localization to CXCL13-rich B-cell follicles within secondary lymphoid tissues (43, 44).

### Effects of the IgG Subclass Composition on the Function of an IgG Antibody Response

“Upstream” IgG3 and IgG1 antibodies exhibit several functional characteristics that endow them with the ability to provide a “first line of defense” against infections, including neutralization of viruses and ADCC of virus-infected cells (Table 2). First, the large hinge region of IgG3 allows greater flexibility of the fragment antigen-binding (Fab) domain, which binds to antigens. Second, the ability of IgG3 and IgG1 Fc domains to bind all FcγRs permits phagocytosis of viral particles *via* FcγRI (the high-affinity FcγR) and FcγRIIa and FcγRIIb (low affinity FcγRs that activate or inhibit phagocytic cell activity, respectively). Finally, binding to FcγRIIc and FcγRIIIa on NK cells initiates ADCC of virus-infected cells, which IgG3 undertakes particularly effectively because it can bind FcγRs in a monomeric form (39). Monocytes express FcγRI, FcγRIIa, and FcγRIIIa and mediate ADCC against tumor antigens (46, 47). However, studies investigating the role of monocyte-mediated ADCC in HIV infection are inconclusive (48, 49), possibly reflecting differences between the assays utilized, and the role of IgG3 and IgG1 antibodies in monocyte-mediated ADCC will not be discussed.

In contrast to IgG3 and IgG1 antibodies, the functional activities of “downstream” IgG2 and IgG4 antibodies are more focused. IgG2 antibodies are particularly effective in binding to antigens with a high epitope density (35), such as polysaccharides, and exhibit Fc domain binding to low affinity FcγRs only, particularly FcγRIIa, which is widely expressed on phagocytic cells (39). The restricted FcγR binding of IgG2 antibodies may reduce antibody-mediated phagocytosis by monocytes (50), which express FcγRI as well as FcγRIIa (51). However, IgG2 molecules exhibit the greatest resistance to proteolytic enzymes, such as cathepsin G (42), which contributes to intracellular proteolysis of pathogens in phagocytic cells, including dendritic cells (52). In contrast, IgG3 molecules are least resistant to proteolysis by intracellular proteolytic enzymes (42). IgG4 molecules are capable of forming antibodies with different Fab domains on each half of the molecule (bispecific antibodies), which decreases the valency of antigen binding, and do not activate the complement system (36). They are often elicited after prolonged antigenic stimulation. These characteristics suggest that IgG4 antibodies play a role in down-modulating the effects of an IgG antibody response (53). Of note, the lack of efficacy of gp120 subunit vaccines in preventing HIV-1 infection may partly be explained by the production of IgG4 antibodies associated with repeated vaccinations (54). The functional activities of the Fc domain of all subclasses of IgG antibodies are strongly influenced by glycosylation of this region of the molecule. HIV-1 infection is associated with a skewing of IgG antibodies to an agalactosylated (G0) glycoform, which is indicative of HIV-induced inflammation of the B-cell compartment (55).

It has been recognized for over 30 years that most viruses, including HIV-1, induce IgG3 and/or IgG1 antibodies to viral antigens with IgG3 antibodies produced first (56). However, IgG2 antibodies are also produced against HIV-1 Env gp41 and Gag p24 and we and others have shown that these antibodies are associated with natural or postvaccination control of HIV-1 infection (19, 57–60). While this might reflect better immunocompetence resulting from greater integrity of GC reactions (32), IgG2 antibodies might contribute to “protective” HIV-1-specific IgG antibody responses, especially those mediated *via* FcγRIIa (59).

### Virus Neutralization by IgG Antibodies

Neutralization of viruses by IgG antibodies prevents infection of uninfected cells by hindering the binding of the virus to cellular receptors, which in the case of HIV-1, is enhanced by the binding of antibodies to FcγRs on phagocytic cells (61, 62). Binding of HIV-1 Env-specific IgG antibodies to FcγRs may lead to the clearance of virion/antibody complexes by macrophages and neutrophils in tissues (63), but this function overlaps with that of opsonophagocytic IgG antibodies that activate phagocytic cells to elicit antiviral responses. Indeed, HIV-1 Env-specific IgG antibodies that exert HIV-1 neutralization activity also induce opsonization of HIV-1 viral-like particles and their phagocytosis by the monocytic THP-1 cell line (64).

### Opsonophagocytic IgG Antibody Responses against Viruses

Opsonophagocytic IgG antibody responses entail the opsonization of antigens by IgG antibodies and phagocytosis of antibody-coated antigens by cells that have the capacity to undertake phagocytosis. Concomitant complement activation may also be involved for some pathogens. Binding of IgG antibodies and complement system components (opsonins) to CRs and/or FcγRs on the surface of phagocytic cells is critical for this process. Binding of IgG antibodies to FcRn also enhances phagocytosis (65, 66). While the effect of opsonophagocytic IgG antibody responses in enhancing the bactericidal activity of neutrophils for bacteria, such as pneumococci (67), is well-characterized, this type of antibody response may also be induced against other types of pathogen, including viruses (Table 1). However, the phagocytic cells and effector functions of those cells are varied, depending on the type of pathogen. In the case of viruses, the most functionally relevant phagocytic cells are cDCs, pDCs, and possibly monocytes, which exhibit significant differences in their expression of FcγRs (39, 51).

Both cDCs and pDCs are able to internalize viruses and bacteria by endocytosis, but the activation and functional effects of these cells are greatly enhanced when antigens are opsonized by IgG antibodies and phagocytosed *via* FcγRIIa (51, 68–70). Targeting of antigens to cDCs by antibodies has been explored as a means of enhancing vaccine-induced CD8^+^ T-cell responses, though not usually with endogenous antibodies (71).

As exemplified by HIV-1, processing of viruses within endosomes of pDCs results in the exposure of viral nucleic acids and their sensing by endosome-expressed toll-like receptors (TLRs) (72). pDCs, but not cDCs, exclusively express TLR7 and 9 (73). TLR7 is activated by single-stranded RNA (ssRNA) in synergy with guanosine or guanosine metabolites (74) and/or guanosine-uridine-rich ssRNA, such as that isolated from HIV-1 (75), whereas TLR9 is a sensor of unmethylated CpG DNA (76). Activation of pDCs by viral RNA results in a “killer” response, whereby pDCs upregulate tumor necrosis factor-related apoptosis inducing ligand (TRAIL), which induces apoptosis of virus-infected cells that express death receptor 5 (77–79). Additionally, activation of pDCs *via* TLR7/9 induces the production of large amounts of type I interferons, particularly interferon-alpha (IFN-α) (80), which upregulate IFN-stimulated genes (ISGs) that encode molecules with multiple antiviral effects (81). Among these are retroviral restriction factors that directly interfere with viral replication (82). IFN-α also enhances NK cell cytotoxicity against virus-infected cells, including HIV-1-infected CD4^+^ T cells (83, 84), and enhances CD8^+^ T-cell clonal expansion by potentiating interleukin-2 signaling (85). pDCs present antigens to CD8^+^ T cells, though not as effectively as cDCs (86).

Interferon-alpha is produced following IgG-mediated opsonophagocytosis of (i) coxsackie B virus, foot-and-mouth disease virus, and classical swine fever virus by pDCs (87–91); (ii) poliovirus and HIV-1 by peripheral blood mononuclear cells (92, 93); and (iii) cocksackievirus B4 by monocytes (94). In the majority of these studies, FcγRIIa was essential for IFN-α production in response to IgG-opsonized virus (87–89, 91, 92, 94), in line with FcγRIIa being the primary FcγR expressed on pDCs, albeit at low levels (51, 95). Although these studies pertain to non-enveloped RNA viruses, it has also been demonstrated in mice that IgG antibodies to influenza virus nucleoproteins provide protection against lethal influenza challenge through a mechanism dependent on FcRs and IFNα/β (96). These findings raise the possibility that capsids of enveloped viruses might also be the target of pDC-reactive opsonophagocytic IgG antibody responses.

### Antibody-Dependent Degradation of Internalized Viruses

The functional effects of an IgG antibody response against viruses may also be exerted intracellularly. An example of this is the binding of the cytosolic Fc receptor tripartite motif containing-21 (TRIM21) to IgG, which occurs with submicromolar affinity (97, 98) making TRIM21 the highest affinity FcR described in humans. TRIM21 recognizes IgG Fc domains on antibodies bound to viruses, through the PRYSPRY domain of the TRIM21 molecule, resulting in E3 ubiquitin ligase activity that destines viruses for proteasomal degradation in the cytosol (99). In parallel, activation of TRIM21 induces a pro-inflammatory and antiviral cytokine response through activation of the transcription factors NFκB, AP-1, IRF3, IRF5, and IRF7 (100). The antiviral function of TRIM21 is augmented in the presence of IFN-α because TRIM21 is IFN inducible (99). In fibroblasts, TRIM21 can be activated by antibodies bound to the surface of pathogens and does not require FcγRs (101). However, transport of antibodies into cells may occur *via* FcRn (20) and a role for FcγRs on phagocytic cells is also possible. The affinity with which TRIM21 binds the Fc domain of IgG3 molecules is lower than for other IgG subclasses (39), suggesting that IgG3 antibodies are less effective in the intracellular degradation of viruses. TRIM21 is critical for antibody-mediated control of virus infections in mice and is also active in human cells (39, 100, 101), though a role in virus infections of humans has not been clearly established.

### NK Cell-Activating IgG Antibodies

In addition to mediating innate immune responses regulated by cell surface activatory and inhibitory receptors, NK cells also act as effector cells for IgG antibodies that bind *via* their Fab domain to viral proteins expressed on the surface of infected cells and *via* the Fc domain to FcγRIIc or FcγRIIIa on the NK cell surface. Activated NK cells mediate ADCC of virus-infected cells and produce immunoregulatory cytokines, such as IFN-γ (102).

## Functional Effects of IgG Antibody Responses Against HIV-1

### Neutralization Activity of HIV-1 Env-Specific IgG Antibodies

While HIV-1 infection can induce Env-specific IgG antibodies with neutralizing activity, these antibodies are often ineffective. HIV-1 exhibits very effective immune evasion strategies, including possession of a “glycan shield” that protects antigenic sites on Env from antibodies, and a pronounced susceptibility of Env glycoproteins to antigenic variation and escape from antibody binding (15). The extent of this problem was aptly demonstrated in recently reported clinical trials of hMAb to the CD4 binding or V3 glycan sites of HIV-1 Env, which demonstrated that neutralization resistance occurred commonly in existent and emergent HIV-1 (103, 104).

Human immunodeficiency virus type 1 infection also causes defects of antibody production that adversely affect the production of Env-specific IgG antibodies. GC dysfunction, including decreased numbers and/or function of T_FH_ cells, is associated with an impaired neutralization activity of Env-specific IgG antibodies in HIV-1 and simian human immunodeficiency virus (SHIV) infection (28, 105–107). Decreased SHM in follicular B cells leading to low avidity of HIV-1 Env-specific IgG antibodies (108) is likely to be a major contributor to this. Indeed, studies of HIV-1 Env gp120 V3 glycan-specific hMAbs have shown that the breadth and potency of HIV-1 neutralization is related to the amount of SHM (109). Furthermore, while HIV-1 Env-specific IgG3 antibodies exert the greatest neutralizing activity, probably related to the longer hinge region of IgG3 (110), IgG3 antibodies to multiple HIV-1 proteins, including Env, decline after acute HIV-1 infection, whereas IgG1 antibodies persist (111, 112). However, broad neutralization activity of HIV-1 Env-specific IgG antibodies increases with longer duration of exposure to HIV-1 infection (113–115), suggesting that at least some of these barriers can be overcome.

### NK Cell-Activating IgG Antibody Responses against HIV-1

A “protective” effect of HIV-1 Env-specific IgG antibodies that activate NK cells has been demonstrated in *ex vivo* experiments (116) and in the RV144 HIV-1 vaccine trial, which demonstrated that the production of such antibodies was the strongest correlate of protection from the acquisition of HIV-1 infection (117). As for virus neutralization, HIV-1 Env-specific IgG1 and/or IgG3 antibodies appear to be sufficient for mediating this type of IgG antibody response against HIV-1-infected cells (54). Interestingly, genetic studies provided evidence that these antibodies bound primarily to FcγRIIc in RV144 trial participants (118). However, HIV-1 Env-specific IgG antibodies with NK cell-activating activity are detected in approximately 80% of patients with active chronic HIV-1 infection (119) and escape by HIV-1 from the effect of these antibodies is substantial (120). Furthermore, HIV-1-specific IgG antibodies that activate NK cells decline after 6 months of infection associated with a decline in IgG3 antibodies to gp120, gp140, and p24 (121). These antibodies may therefore be less effective in controlling HIV-1 infection than in preventing the acquisition of HIV-1 infection.

### Opsonophagocytic IgG Antibody Responses against HIV-1

Emerging evidence suggests that opsonophagocytic IgG antibody responses against SIV or HIV-1 virions, and possibly HIV-1 capsids, may contribute to the control of SIV or HIV-1 infection by enhancing the effector functions of cDCs and pDCs. Studies undertaken in macaques with early SIV infection have shown that infusions of acute phase SIV Env-specific IgG antibodies with neutralizing activity suppress SIV replication for up to 2 years and that this effect is associated with an increased uptake of SIV by cDCs and an enhancement of SIV-specific CD4^+^ and CD8^+^ T-cell responses (12, 122, 123). Enhancing HIV-1 uptake by cDCs is also a possible explanation for the observation that a single infusion of the HIV-1 Env CD4 binding site (CD4bs)-specific hMAb 3BNC117 augmented production of HIV-1 Env-specific IgG antibodies with neutralizing activity (11).

Activation of pDCs by opsonophagocytic IgG antibodies might also contribute to the control of SHIV or HIV-1 infection. Clearance of SHIV from infected tissues of macaques by a HIV-1 gp120 V3 glycan-specific hMAb (PGT121) was shown to be associated with an increased production of multiple ISGs at sites of SHIV infection (124), suggesting that the antibodies mediated opsonophagocytic activity that activated IFN-α-producing cells, which are likely to have included pDCs. Also, we have provided evidence that pDC-reactive opsonophagocytic IgG antibody responses against HIV-1 Gag proteins (e.g., p24) are associated with the control of chronic and early HIV-1 infection (18, 19) and argued that these findings might explain the numerous historical reports that IgG antibodies to HIV-1 Gag proteins are associated with slow progression of HIV disease [reviewed in Ref. (125)].

A greater understanding of the characteristics of both IgG antibodies and FcγRs associated with “protective” opsonophagocytic IgG antibody responses against HIV-1 virions or capsids will be required if hMAb therapy or vaccine-induced IgG antibodies are to be used to control HIV-1 infection. To date, FcγR-mediated antibody function has been measured using high-throughput assays that have yielded valuable insights into protective antibody responses following vaccination or natural infection. However, given that these assays are limited to assessing one type of FcγR-mediated function for one cell type at a time, they provide an incomplete picture of antibody function *in vivo*, which is multifaceted (126). Examination of antibody characteristics associated with the opsonization of HIV-1 virions by various isotypes of antibodies to various HIV-1 Env antigens, and their phagocytosis by a monocyte cell line (THP-1 cells), demonstrated that IgG3 antibodies were more effective than IgG1 antibodies and that plasma IgA1 and IgA2 antibodies were inhibitory (127). However, monocytes use FcγRIIc and FcγRI to a greater extent than cDCs and pDCs (51) and while examining the opsonization of HIV-1 virions by Env-specific IgG3 antibodies and their phagocytosis by monocytes may elucidate mechanisms by which vaccine-induced IgG antibodies prevent the acquisition of HIV-1 infection (128), this may not be so for control of chronic HIV-1 infection. Monocyte-reactive opsonophagocytic IgG antibodies to HIV-1 gp140 (disulfide-stabilized trimers of gp120 and the ectodomain of gp41) are produced during early HIV-1 infection, but it is unclear if they are associated with the control of HIV-1 replication, and the partially successful vaccination strategy used in the RV144 trial did not induce these antibodies (129).

### HIV-1-Specific IgG Antibodies in HIV Controllers

Investigations of HIV-1-specific IgG antibodies in patients who control HIV-1 infection without the use of ART and maintain plasma HIV RNA levels of <50 copies/mL (elite controllers) or 50–2,000 copies/mL (viremic controllers) have the potential to provide information about antibody-mediated control of HIV-1 infection. However, studies thus far have not led to clear conclusions. Plasma levels of HIV-1 Env-specific broadly neutralizing antibodies, including those that bind the gp120 CD4bs, gp41, or Env epitopes bound by the hMAbs 2F5 and 1b12, are variable among elite and viremic HIV controllers and do not correlate with HIV-1 viral load (130, 131). Some studies have shown that HIV-1 Env-specific antibodies with ADCC activity were higher in elite controllers than in non-controllers (58, 130, 132); however, one study found no difference (133). Monocyte-reactive HIV-1 gp120-specific opsonophagocytic antibodies are similar in HIV controllers and non-controllers (134). However, while the magnitude of individual HIV-1 Env-specific IgG antibody effector functions is similar in HIV controllers and non-controllers, HIV controllers are distinguished by their ability to elicit a combination of, or “polyfunctional,” IgG antibody effector responses against HIV-1 gp120, i.e., activation of, and cytokine production from, multiple FcγR-bearing cells (17).

We have shown that plasma HIV-1 p24-specific IgG2 and/or IgG1 antibody levels and pDC-reactive opsonophagocytic IgG antibody responses were associated with the control of HIV-1 replication in viremic patients, particularly controllers (19), and in patients with early HIV-1 infection (18). In contrast, higher HIV-1 gp140-specific IgG2 antibody levels were present in elite controllers (19), supporting the findings of previous studies (57, 60, 135). One interpretation of these findings is that HIV-1 p24-specific pDC-reactive opsonophagocytic IgG antibody responses contribute to the control of HIV-1 infection in individuals with active HIV-1 replication, while HIV-1 Env-specific IgG2 antibodies are a marker of immune responses associated with elite control. Further studies are needed.

## Effects of HIV-1 Infection and ART on HIV-1-Specific IgG Antibody Responses

Human immunodeficiency virus type 1 infects or affects cells within GCs of lymphoid tissue early in the infection and reservoirs of HIV-1 infection are established in FDCs and T_FH_ cells. These reservoirs persist in patients receiving ART (136–140), in part due to ART penetrating poorly into lymphoid tissues (141). The inflammatory response generated initially causes follicular hyperplasia but subsequently involution occurs (142). Destruction of GC architecture by HIV-1 replication disrupts the interaction between T_FH_ cells and GC B cells, causing an impairment of antibody responses to HIV-1 and other antigens (137, 143, 144). HIV-1 infection causes T_FH_ cell dysfunction (140, 144, 145), which may adversely affect the control of HIV-1 replication. In one study, HIV patients exhibiting HIV-1 Env-specific IgG antibodies with broad neutralization activity had a higher frequency of peripheral CXCR5^+^PD-1^+^CD4^+^ T_FH_-like cells compared to patients who exhibited antibodies with narrow neutralization breadth (106). They also produced greater amounts of class-switched antibodies. Introduction of ART during early HIV-1 infection can restore the architecture of lymphoid follicles (146), though it is unclear whether GC function is improved.

Circulating B cells of individuals with HIV-1 infection are also dysfunctional (147–150) and this persists, albeit it to a lesser degree, on ART (151). However, there is currently no evidence to suggest that this adversely affects the production of HIV-1 Env-specific IgG antibodies with broad neutralizing activity (152) or of IgG antibodies to other antigens (151).

Increased plasma levels of IgG antibodies to multiple HIV-1 proteins and glycoproteins are observed for up to 40 weeks post-infection (153). The IgG antibody response against all HIV-1 antigens is dominated by IgG1 antibodies with IgG3 antibodies declining during primary HIV-1 infection (111, 112, 154). There is little change in plasma levels of total IgG antibodies to multiple HIV-1 proteins during chronic HIV-1 infection, even in patients who develop AIDS (112), unless ART is commenced. Commencement of ART during early HIV-1 infection leads to a decline in plasma levels of IgG antibodies to multiple HIV-1 antigens (153, 155) with plasma levels of HIV-1 p24-specific IgG1 and IgG2 antibodies showing the closest relationship with viral replication (154, 156). Introduction of ART in patients with very early HIV-1 infection who have not produced HIV-1-specific IgG antibodies may prevent the production of those antibodies (157). When considered together, the findings of these studies indicate that the production of HIV-1-specific IgG antibodies is dependent on HIV-1 replication and declines during ART.

Data on the effect of ART on the function of HIV-1 Env-specific IgG antibodies are limited. Introduction of ART results in a decline in HIV-1 Env-specific IgG antibodies that elicit NK cell activation or opsonization of HIV-1 gp140-coated beads and phagocytosis by monocytes (THP-1 cells) (158). With regard to neutralization activity, it has been reported that all HIV patients receiving long-term ART exhibit plasma HIV-1 Env-specific antibodies with neutralizing activity against at least one heterologous HIV-1 (159). However, ART does not usually increase HIV-1 Env-specific antibodies with neutralizing activity against autologous HIV-1 (160, 161). The relationship of HIV-1 Env-specific IgG antibody avidity with the control of HIV-1 infection, and the effect of ART on this, is unclear. While higher avidity of Env-specific IgG antibodies has been associated with the control of SHIV infection in macaques (162), a relationship between antibody avidity and control of HIV-1 infection has not been clearly established. This partly reflects uncertainties about the validity of antibody avidity indices determined by some assay methods (163). Furthermore, some studies have demonstrated that ART decreases the avidity of HIV-1-specific antibodies in patients with primary HIV-1 infection (164) while others have demonstrated that it has no effect (154, 165). This likely reflects that different assay methods perform differently in the context of ART (166). Interestingly, the use of intra-vaginal tenofovir gel prophylaxis in women who subsequently became infected with HIV-1 was reported to slow the maturation of antibody avidity (167).

## Future Research on Therapeutic Approaches to Enhancing HIV-1-Specific IgG Antibodies, Including Therapeutic HIV Vaccines

Recognition that IgG antibodies may contribute to the control of HIV-1 infection has reinvigorated research on HIV-1-specific IgG antibody responses, particularly the functional characteristics of antibodies associated with durable control of HIV-1 replication, as well as research on prevention and/or correction of defective IgG antibody responses caused by HIV-1 infection. Introducing ART as early as possible should preserve the structure and function of GCs and enhance IgG antibody function by optimizing SHM, affinity maturation, and immunoglobulin isotype diversification in the follicular B cells that differentiate into plasma cells producing HIV-1-specific IgG antibodies. In addition, strategies to optimize antiretroviral drug levels in lymphoid tissue may be beneficial (168). However, as ART decreases the antigenic stimulus for the production of HIV-1-specific IgG antibodies by reducing HIV-1 replication, interventions to boost antibody production will be required, including vaccination, especially for “shock and kill” strategies designed to decrease the size of HIV-1 reservoirs. The generation and maintenance of vaccine-induced IgG antibody responses is likely to depend on the integrity of the GC reaction, and it may therefore be informative to include the assessment of GC function when developing strategies for the evaluation of HIV-1 therapeutic vaccines. Preliminary data suggest that plasma CXCL13 levels and production of circulating T_FH_ cells after vaccination might have clinical utility (144, 169).

To enlighten the characteristics of HIV-1-specific IgG antibody responses that should be generated by therapeutic HIV-1 vaccines, future studies in HIV controllers should determine if multifunctional HIV-1-specifc IgG antibody responses are most effective in controlling HIV-1 infection and which antibody functions and effector cells should be targeted. In addition to exerting neutralization activity, IgG antibodies that activate effector cells with antiviral activity, specifically cDCs, pDCs, and possibly NK cells, would appear to be the most appropriate targets. In undertaking these studies, consideration should be given to the likelihood that the functional effects of antibodies are most apparent in patients with active HIV-1 replication and to the possibility that opsonophagocytic and intracellular IgG antibodies might also target HIV-1 capsids.

Given that the IgG subclass content of HIV-1-specific IgG antibody responses affects antibody function, the efficacy of therapeutic HIV-1 vaccines might be enhanced by the administration of vaccine antigens with adjuvants or cytokines that not only enhance the magnitude of the antibody response but also change the IgG subclass content. In non-human primates (NHPs), IgG2 and, to a lesser extent, IgG3 antibodies to *Leishmania donovani* antigens are enhanced when vaccination is undertaken with alum-BCG or montanide ISA720 (170), and in mice, vaccination with HIV-1 virus-like particles adjuvenated with poly(I:C) or CpG ODN1826 changes the IgG subclass content of both Env- and Gag-specific IgG antibody responses (171). Furthermore, recombinant GM-CSF has been used to enhance the uptake of the Vacc-4x HIV-1 p24 vaccine by DCs in a “shock and kill” strategy that reduced the size of the HIV-1 reservoir (172), though the effector immune responses have not been reported, and local IFN-γ production may have enhanced production of HIV-1 Gag-specific IgG2 antibodies by a DNA vaccine that was associated with the control of HIV-1 replication after ceasing ART (59, 173). The effect of adjuvants or cytokines on the IgG subclass content of vaccine-induced SIV or SHIV Env-specific IgG antibody responses could be assessed in NHPs and, furthermore, clinical studies of therapeutic HIV-1 vaccines given with adjuvants or cytokines might include an analysis of the IgG subclass content of HIV-1-specific IgG antibodies. Finally, as suggested by Figure 1, vaccination strategies that enhance combined cellular and humoral HIV-1-specific immune responses may be required to control or eradicate HIV-1 infection and should be investigated.

## Author Contributions

All authors contributed to writing the article with MF taking the leading role. The final version of the manuscript was reviewed and approved by all authors.

## Conflict of Interest Statement

The authors declare that the research was conducted in the absence of any commercial or financial relationships that could be construed as a potential conflict of interest.
